# American Dental Society of Europe

**Published:** 1874-03

**Authors:** C. M. Wright


### Proceedings of Societies.

### "AMERICAN DENTAL SOCIETY OF EUROPE."

*Reported by* C. M. WRIGHT.

The semi-annual meeting of this wide-awake little Society met in the offices of Drs. Van Marter and Wright, in Basle,

Switzerland, on the 27th of November, 1873, and continued its session two days.

A Constitution and Order of Exercises were adopted. The only distinctive feature of the Constitution is, the qualification of members : "The active member shall be a resident practicing physician of Europe, who holds a diploma from a recognized American dental college and who is recommended by the Committee on Membership, or one who shall pass a satisfactory examination before the Committee on Membership.

"The honorary members shall consist of practicing dentists of repute, not American citizens, who shall apply for membership and are acceptable to the Committee on Membership ; also, of distinguished American and foreign dentists, physicians and surgeons who render assistance and encouragement to our cause ; etc."

The meeting was opened by appropriate religious exercises by the Rev. Mr. Nielson, of the American Mission Church.

The President, Dr. Terry, of Zurick, delivered a short address, mentioning the objects of our Society as, 1st, advancement in professional knowledge, and, 2d, acquaintance with each other as dentists and American citizens. He spoke of the need of a society so long felt here in Europe, and of the encouraging prospects of our young society—urged harmony in our discussions, and remarked that as all dentists are not equally gifted, one can talk, another can do, etc. The address was followed by the following welcome from Dr. Van Marter:

"Mr. President and Gentlemen:—We have felt that the Am. Dental Society of Europe, while still in infancy, should be encouraged with kind words and cheerful acts until it has arrived at that age when it can go alone. As a little child, in its first efforts in the difficult gymnastic feat of walking across the room, often needs the coaxings, fondlings and encouragements of its mother, so we have felt our little society in its totterings and tumbles and unsteadiness, so natural in one so young—needs fondlings and encouragements, and having the paternal or maternal feelings in our breasts for the little one, wish to welcome it in its second effort, and to express our happiness in receiving the child of our suggestion in Basle, with open arms and 'God bless you.' Welcome! gentlemen of the Am. Dental Society of Europe to Basle!

Our number is small at present, but when we know the energy, the intelligence, the *good blood* that courses the veins of this Society, can we not count on a future of success, a future of greatness, a future of honor? When I speak of good blood, I mean our rich American blood, and the sinews we received from our Alma Mater.

Every great undertaking began with days of small things—Every American Dental Society began with a few earnest members. Every dental college began with what we have here—energy and determination to succeed, no matter what clouds appear in the horizon.

Our Society, gentlemen, has begun with many encouraging signs. Our sky is tolerably clear, and the number of letters from American dentists in Europe—from France, Germany, Italy, Austria and Switzerland, that we have received show the interest already excited in our professional brothers' minds, about our undertaking. Every letter is one of encouragement and an offer of assistance and hope. And from the data we have, I think we can promise without too much of the element of hope entering into our calculation, that in one or two years, this Society will rank with our older sister societies in our much loved America. Before that time, we hope for recognition by the American Dental Association, and the opportunity of sending our accepted delegates to that honorable body.

Again, we welcome you to Switzerland, and believe you will agree with us, that it is not only auspicious but very appropriate, that our infant Society should be rocked in the cradle of Swiss liberty, ere it tries its powers in other lands. Let us then, as Lock says, 'Seek the sharp encounter of mind with mind, yet not that any energy or power may be curtailed or rendered less effective, but rather that, as in the encounter of flint with steel, light may be evoked where obscurity prevailed before.' "

After the address the following subjects for discussion were offered by the Executive Committee and accepted.

SUBJECTS FOR DISCUSSION.

1. Associated effort, its influence upon the development and progress of our profession.

2. Filling of children's teeth, should it be permanent or temporary?
3. Rubber dam and appliances. Use of Matrix.
4. Does the bur, as used in Burring engine, injure the tooth substance?
5. Uniform fee bill.
6. Contour fillings.
7. Opening of pulp cavity and preparation of the roots for filling.
8. Preparation of cavities.
9. Treatment of alveolar abscess.

The first subject was opened by the following paper by Dr. N. W. Williams, of Geneva. [See article on 114th page of this No.]

A short discussion followed.

The second subject, "Filling of children's teeth—should it be temporary or permanent?" was opened by Dr. de Trey, of Vevay:—

"There is a great difference in the texture of children's teeth. Favors temporary fillings, unless the teeth are of *especially* good quality and texture. At the age of twelve would fill permanently; before that age prefers to use os-artificial, which he mixes as dry as possible. Has had better success with this material used in this way, both as regards the avoidance of pain, which is an important matter in the treatment of children's teeth, and also as regards the durability of the artificial bone. Has not had experience in the use of amalgam, therefore is not yet satisfied whether the material is good for these cases or not. Sometimes before the teeth are fully developed caries attacks them: thinks it not advisable to use gold in these cases, as the secretions during this period have an acid re-action and the teeth will often require refilling; therefore favors the temporary stopping. In regard to using Hill's Stopping and Guilloi's Cement, pursues the following indica tions: Alkalines affect the Hill's Stopping—acids, the Guilloi's Cement. In mouths where alkaline re-action occurs, the Hill's Stopping becomes brittle; in acid mouths the Guilloi's is partially dissolved. Has watched and tested with litmus paper this condition in very many mouths for several years, and now before using either he applies his litmus paper, testing the secretions and deciding according to the above conclusions whether he shall use Hill's Stopping or Guilloi's Cement in the particular case before him. Has had sufficient evidence, he thinks, to warrant him in urging the test-paper method on every member."

Dr. Van Marter, of Basle, considered the question as referring to the temporary teeth. In filling these teeth had found Hill's Stopping too temporary a filling often, in large cavities, and prefers in most cases tin and amalgam. When amalgam is employed, uses it very dry and burnishes the surface with layer upon layer of tin foil. The tin foil absorbs still more of the mercury, leaving a good tin surface. This practice he regards as good in all cases where amalgam is used. Sometimes fills the bottom of a cavity with tin foil and then covers with amalgam, treating the surface as before stated. Related experience with his own child, in whose mouth he had tried Hill's Stopping, the file, Guilloi's Cement and finally amalgam in the same cavities. Objects to the use of Guilloi's for children, where the cavity is deep, because of the pain so often caused, and the bad policy of causing pain in little patients who do not understand the necessity of these operations. In the six year old molars, fills with gold in nearly all cases and tries to teach the little patients the necessity of cleanliness, also requires them to be presented at least every six months. In case of neglect on the part of the parents, does not hold himself responsible. Where the teeth are of a chalky quality, gives lime water internally, in teaspoonful doses daily. In very many cases has found the quality improve. Thinks different localities offer different circumstances and, consequently, we frequently talk at cross purposes, because we don't understand *all* the circumstances. Certain peculiarities prevail in certain sections. Certain modes of practice will be good here and bad at home, and bad even in a different climate here in Europe.

Dr. Field, Geneva. Until within two years believed that gold was the only material for filling everything presented, even temporary teeth ; but, on account of many failures, has abandoned this practice. In temporary teeth fills with Hill's Stopping, or amalgam mixed *very dry*; and in very soft teeth, where the caries has been extensive, uses Guilloi's, applied as carefully as for an adult, using the coffer dam and covering the filling with melted wax and allowing it to remain for half an hour before removing the dam. In six year molars prefers never to use gold before the age of fifteen for his patients, excepting where the teeth are of a noticeably good and firm texture. In other teeth for children prefers never to use gold till the age of twelve, unless, as before, a marked firmness of quality warrants the operation (and in this country this is rare).

Refuses to operate for children sometimes, when the parent neglects to bring them often for examination and consultation. Where we have the control of the patient, can employ the best materials, and re-apply them if necessary, and are doing often the best thing for them. Where we have not control, had better not operate; for our best gold work may only be temporary, and reputation suffer.

Dr. Terry, Zurick, has been practicing in this part of Europe ten years, and has watched as faithfully as possible the results of his own work, and believes in using gold in the permanent teeth of children—thinks it is the best filling in a large majority of cases. In temporary teeth uses Hill's Stopping and thinks one great reason why this material fails so often is overheating before applying. Also uses in masticating surfaces amalgam. Mentioned a case he had lately seen, where amalgam, Guilloi's, and Hills Stopping had been put in the mouth of a child at the same time; the amalgam alone was preserving the teeth. In some cases he has used in approximal cavities in children's and adult's teeth tin foil at the bottom of the cavity, next to the gum, and Guilloi's for the rest of the cavity, with good results.

Dr. Williams, Geneva, thinks the question of locality an important one. Has had greater difficulty in saving the teeth of Geneva children than at home in Ohio. For some children it is almost impossible to make good gold fillings, and here he prefers some plastic filling. In front teeth uses Hill's and in molars and bicuspids prefers tin foil though often this requires as much skill as gold to make it right. This he considered good practice in regard to the permanent teeth of children in America. Here, he uses more Guilloi's. In large cavities employing a layer of Hill's stopping over the dentine to protect it from the action of the Guilloi's. Mixes Guilloi's so that in consistency it is like Hill's stopping when ready to use.

In very favorable cases, uses gold foil for filling, and especially when we can not see the patient as often as we wish. You have all noticed Hill's filling badly cupped in the center, while the edges are quite full and the tooth protected.

Dr. Wright, Basle, said he should like to talk, but as it is impossible to write and talk, he should consider his duty done if he recorded faithfully the interesting remarks of the learned gentlemen present,—and in making this record he should take the liberty of condensing into one speech all that the gentemen say in two or three possessions of that proud place—"the floor."

The next subject was called for and discussed.

### RUBBER DAM AND APPLIANCES.

Dr. Field uses the dam in nine cases in ten—in nearly all cases, in fact sometimes even where it is unnecessary for dryness—but the freedom from all anxiety its use gives is valuable and the habit of its use is fascinating. The old method of napkin and bibulous paper even when successfully used for the prevention of moisture was always accompanied by anxiety lest some accident might allow the enemy of adhesive foil to come in on the half finished filling. Uses the ordinary appliances —waxed thread, wedges, clamps, etc.—as a proper or even fair use of the rubber dam implies a use of the appliances accompanying it. Asked if we remembered the old method of filling with gold a large approximal cavity—and asked us to compare the present method.

Dr. Williams:—"The more we use the rubber dam the more valuable it becomes, and the more we feel like using it." At home used it much more sparingly than at present. In many cases it is unpleasant to the patient and in some cases where he feels quite certain that he can get along without the dam, does not use it, and especially if there is difficulty in applying it. Finds some cases where it is impossible for him to apply it.

Dr. de Trey also uses the dam in nearly every case and regards it one of the most useful of modern appliances. Mentioned that formerly he used soda for cleansing the rubber after use, but found that the strong alkaline made his rubber brittle. In some cases where the gum overlaps the borders of cavities, uses the actual cautery to clear it away.

Dr. Terry considers the rubber dam one of the luxuries of dental practice. and has thus far met with only one patient who from nervous excitability complained so much of choking and difficulty in breathing that he was compelled to leave off the dam. Uses it sometimes under a napkin to prevent moisture, and at the same time overcome the difficulty of light on account of the dark color of the rubber. In applying the dam for approximal cavities where the cervical wall is difficult of access he cuts through the gum with an excavator, and drives a wedge deep down on the gum between the teeth.

Dr. Van Marter can't see how a man can apply the coffer dam in nine of the cases in ten which are presented to us for filling, nor can he believe that in the great majority of cases the rubber dam can be used with advantage or comfort to either operator or patient. Of course he acknowledges with pleasure the value of the dam in certain cases—for there are places where it is almost invaluable, and he uses it with much satisfaction in these cases, but does not ride the hobby. In this locality, there are presented many simple cavities to be treated, where the application of the rubber dam would be a waste of time, and the napkin and bibulous paper are all sufficient, and much more agreeable to the patient—insisted that though the dam is a very good instrument or appliance, yet discrimination is necessary to its proper use. We must look to the comfort of our patients as well as to our convenience, provided the excellence of our work is not affected.

"Does the burr, as used in the Burring Engine, injure the tooth substance?"

Dr. Van Marter thinks it might be considered an axiom, that if the burr is used properly and at not too great speed it can not injure the tooth substance; uses Morrison's engine daily, and has seen no ill or deleterious effect on tooth substance.

Dr. de Trey is very particular about keeping the tooth wet while using the burr, and especially in finishing filling—uses a drop tube with water to keep the burr cool; considers the coarse burrs objectionable, and prefers even the finishing burrs as better adapted for cutting tooth substance.

Dr. Terry runs the engine rapidly, but don't hold the burr on any one place too long; thinks pain is avoided by rapidity of motion, on the principle that a sharp excavator causes less pain than a dull one; is very partial to corundums in various shapes adapted to the mandrills of the engine.

Dr. Williams runs the engine also at high speed, applying the burr like a chisel to scoop out a portion of tooth substance rapidly and painlessly, and has certainly observed no injurious result to tooth substance; objects to preparing cavities under water or saliva; keeps them dry in the preparatory work.

Dr. Field has had a patient who came from a distinguished European American dentist, who told him that the former operator insisted that the burr caused great injury to the tooth, that he, the American dentist never used it except for finishing fillings; that was why he wished this subject discussed, as he himself had never before thought of injury resulting from the burr.

Dr. Wright thought if injury does result it must be on account of the heat engendered; this implies the incautious use of this valuable instrument, and this could as easily follow the finishing of metal fillings as from boring on dentine, as the thermal effects would be rather increased by the conductability of the metal filling; thinks any instrument can be made to injure the teeth—the steel mallet though a very nice instrument might be used in breaking off the front teeth, and yet no one would question its proper use.

The next subject on the list, "Uniform Fee-bill," was discussed in an informal manner. Some of the members favoring a uniformity in the minimum charges, and others considered such a fee-bill as impossible, as location, class of patients, etc., etc. affected the charges.

The subject of "Contour Fillings" was next before the Society. Essays were read by Dr. G. W. Field, of Geneva; and Dr. C. M. Wright, of Basle. Some discussion followed; some favorable to and others opposed to the operation.

Dr. Van Marter criticised the statements that are said to come from Prof. McQuinlan, in the Southern Association, in regard to the young man who had a few teeth filled with gold, and from that point was carried to Paris, and there made his millions; thinks probably the report did not do Prof. McQuinlan justice. Many a young man has wasted gold in beautiful contour operations in the laboratory; has carried them in his pockets, has displayed them in dental societies much to the annoyance of older practitioners, and has remained so poor that his nails won't grow. Some of these young men have even come to Paris and are still poor. This is no evidence for or against contour operations.

As time was limited, only the papers on the following subjects for discussion were presented, viz: One from Dr. Williams on the Preparation of Cavities, and one from Dr. Kingsley, of Paris, on "Opening of Pulp Cavity and Preparation of the Roots for Filling,"

The Society then adjourned to meet July 2d and 3d, 1874. A cordial invitation is extended to American brothers traveling in Europe next summer to make their route in accordance with the meeting, as we have a live Society and interesting times—and a welcome for our professional friends.

